# Cancer Mortality in Residents of the Cadmium-Polluted Jinzu River Basin in Toyama, Japan

**DOI:** 10.3390/toxics6020023

**Published:** 2018-04-06

**Authors:** Muneko Nishijo, Hideaki Nakagawa, Yasushi Suwazono, Kazuhiro Nogawa, Masaru Sakurai, Masao Ishizaki, Teruhiko Kido

**Affiliations:** 1 Department of Public Health, Kanazawa Medical University, Uchinada, Ishikawa 920-0293, Japan; 2 Health Evaluation Center, Kanazawa Medical University Hospital, Uchinada, Ishikawa 920-0293, Japan; 3 Medical Research Institute, Kanazawa Medical University, Uchinada, Ishikawa 920-0293, Japan; hnakagaw@kanazawa-med.ac.jp; 4 Department of Occupational and Environmental Medicine, Graduate School of Medicine, Chiba University, Chiba 260-8670, Japan; suwa@faculty.chiba-u.jp (Y.S.); nogagwa@chiba-u.jp (K.N.); 5 Department of Hygiene, Kanazawa Medical University, Uchinada, Ishikawa 920-0293, Japan; m-sakura@kanazawa-med.ac.jp (M.S.); issa1@kanazawa-med.ac.jp (M.I.); 6 Department of Community Health Nursing, School of Health Sciences, Kanazawa University, Kanazawa 920-0942, Japan; kido@mns.mp.kanazawa-u.ac.jp

**Keywords:** cadmium, follow-up study, cause of death, mortality, environmental pollution, cancer

## Abstract

After 26 years, we followed up 7348 participants in a 1979–1984 health screening survey in the Jinzu River basin, the heaviest cadmium-polluted area in Japan. We assessed the associations of cadmium exposure levels and mortality from cancer and renal damage, indicated by records of proteinuria and glucosuria in the original survey. Mortality risks (hazard ratios) were analyzed using the Cox proportional hazards model, stratified by sex, after adjusting for age, smoking status, and hypertension, as indicated in the original survey records. In men, the adjusted hazard ratio for mortality from lung cancer was significantly lower in individuals residing in an area of historically high cadmium exposure and in subjects with a historical record of proteinuria, glucosuria, and glucoproteinuria. The risk of mortality from prostate cancer was borderline higher in cadmium-exposed men. In women, historical cadmium exposure was not associated with an increased risk of mortality from malignant neoplasms, but the adjusted hazard ratios for death from total malignant neoplasms or from renal and uterine cancers were significantly higher in exposed subjects with a historical record of proteinuria, glucosuria, and glucoproteinuria. These findings suggest that women residing in cadmium-polluted areas who exhibit markers of renal damage may be at risk of dying of cancer.

## 1. Introduction

Cadmium (Cd) compounds have been classified as human carcinogens by the International Agency for Research on Cancer [1], leading to studies of mortality causes in Cd-exposed populations. Nawrot et al. (2006) reported a significant association between Cd exposure and lung cancer risk in a Belgian cohort, suggesting that aspiration of house dust containing contaminated soil particles may be related to an increase in the incidence of lung cancer [2]. In male factory workers exposed to high levels of Cd, increased incidences of prostate cancer were observed in Sweden [3] and the UK [4]. An increased breast cancer risk was reported in US women with higher urinary Cd levels [5] and in Swedish women whose dietary Cd intake was high [6]. Akesson et al. (2008) reported that dietary Cd intake increased postmenopausal endometrial cancer incidence in a Swedish cohort [7]. In the general American population, increased mortality from lung and pancreatic cancers in men, and from ovarian and uterine cancers in women, were suggested to be associated with urinary Cd [8]. Also, a significant association between Cd exposure and renal cancer has been reported [9].

In our previous study in residents of the Cd-exposed Jinzu River basin in Toyama, Japan [10], the mortality risks for cancers of the colon and rectum, uterus, and kidney and urinary tract were significantly higher in exposed women with glucoproteinuria. These findings suggested increased cancer risks associated with renal damage induced by Cd exposure. The small sample, however, precluded an analysis of mortality risks for specific cancers.

In this study, we investigated the associations between Cd exposure and mortality risks for specific cancers, and analyzed mortality risks in Jinzu River basin residents with historical records of renal damage (indicated by proteinuria, glucosuria, and glucoroteinuria).

## 2. Materials and Methods

### 2.1. Study Subjects

The Jinzu River basin, the largest and most Cd-polluted area in Japan, is an endemic area of itai-itai disease, which is prevalent among older women and characterized by osteomalacia with severe bone pain and renal tubular dysfunction [11,12]. Cd exposure in this area was divided into five levels (no exposure and borderline, mild, moderate, and high exposure) based on the contribution of contaminated Jinzu River water to irrigation water in each area. The prevalence of itai-itai disease or glucoproteinuria in women aged over 50 years has previously been shown to be associated with Cd exposure [13]. After the discovery of itai-itai disease, the Japanese government conducted health screenings in six Cd-polluted areas, including the Jinzu River basin, to identify residents with renal damage.

A total of 7348 participants (3363 men and 3985 women) in the 1979–1984 health screening survey in Toyama, who constituted 97.6% of the 7531 residents aged over 50, were targeted in the present follow-up survey. These participants lived in the Cd-polluted Jinzu River basin areas of Toyama City, Fuchu, Ohsawano, and Yatsuo. Non-polluted sections of two towns and five cities were selected as controls, and a total of 2098 residents (926 men and 1172 women) participated in the health screenings. These controls were also used in the present survey. Table 1 shows the age distribution, smoking status, and hypertension among the exposed and control subjects, obtained by questionnaire during the original survey [14]. The participants in the exposed areas were divided into two groups: the renal dysfunction group with proteinuria, glucosuria, and glucoproteinuria (807 men and 801 women), and the healthy resident group with neither glucosuria nor proteinuria (2556 men and 3184 women).

After the first step of the baseline health screening test, urinary Cd and urinary beta2-microglobulin (β2-MG) were measured in the subjects with proteinuria, glucosuria, and glucoproteinuria. Table 2 shows medians with the 90–95th of urinary Cd and positive rate (%) of urinary β2-MG in groups with different urinary findings: only glucosuria, only proteinuria, glucosuria and proteinuria. The 95th percentiles of urinary Cd of all groups were more than 20 (μg/L), indicating that the subjects with any urinary findings were highly exposed to Cd. In addition, the rates of increased urinary β2-MG were more than 25% in all groups, except for the male glucosuria group. Particularly in women, the β2-MG positive rate was high (36.5%), even in the glucosuria group. These results suggest that not only groups with both glucosuria and proteinuria but also groups with only glucosuria or proteinuria include subjects with renal tubular dysfunction induced by Cd.

### 2.2. Follow-Up Survey

After obtaining permission to use family registry records for scientific purposes from the regional Legal Affairs Bureau in June of 2005, we collected the registry records of all subjects from each city office and determined their survival status (alive or dead) as of 30 November 2005. Dates and causes of death were determined from vital statistics data after receiving permission from the Ministry of Health and Labor to use vital statistics for research purposes on 12 August 2009. One hundred and sixty-six subjects (1.7%; 133 exposed and 33 control subjects) were excluded because their status could not be ascertained. A total of 5351 deaths were recorded in the exposed and control cohorts, and causes of death for 5276 cases (98.5%) were determined from the records. Individual causes of death were classified according to the Ninth Revised International Classification of Diseases (ICD 9) in the 1979–1994 survey and ICD 10 in the present survey.

### 2.3. Mortality Analysis

To determine the survival period of each subject, the date of the original health survey was considered the starting point, and 30 November 2005 was considered the end of the follow-up period. A total of 232 subjects, including 157 cases with unknown life status and 75 cases with unknown causes of death, were excluded from the analysis. A mortality risk (hazard) analysis was conducted after adjustment for age, smoking status during the original survey period, and history of hypertension, using the Cox proportional hazards model stratified by sex. Hazard rates were compared between exposed and control subjects, among exposed subjects with different exposure levels, and between subjects with and without urinary findings (glucosuria or proteinuria) during the original survey period. The analyses were performed using SPSS software (Version 21.0, IBM, Armonk, NY, USA, 2012). *p* < 0.05 was considered statistically significant.

## 3. Results

### 3.1. Dose–Effect Relationships between Cd Exposure Levels and Prevalence of Proteinuria and/or Glucosuria

We used glucosuria and proteinuria as markers of renal damage induced by Cd, although glucoproteinuria (combined glucosuria and proteinuria) is commonly used as the marker in epidemiological surveys. Therefore, before the mortality analysis, we assessed the associations between these markers and Cd exposure levels in both sexes using logistic regression after adjusting for age, smoking status, and history of hypertension (Table 3). The adjusted odds ratios of having urinary findings of proteinuria, glucosuria, and glucoproteinuria were significantly higher in the Cd-exposed cohort than in the controls for both sexes. Also, the adjusted odds ratios were significantly higher in men in the high-exposure group than in controls. In women, the odds ratio of having urinary findings of proteinuria, glucosuria, and glucoproteinuria increased as the exposure level increased, with significantly higher odds ratios in the mild-, moderate-, and high-exposure groups. These findings suggest that proteinuria, glucosuria, and glucoproteinuria are biomarkers of Cd effects on renal function in both sexes, but the association was higher in women.

### 3.2. Comparisons of Cancer Mortality Among Areas with Different Cd Exposure Levels

Table 4 displays the adjusted hazard ratios for mortality from all and from specific malignant neoplasms of all subjects in the Cd-polluted and control areas. No significant increase in the adjusted hazard ratio was found for deaths from malignant neoplasms in either sex. In men, the highest adjusted hazard ratio in the polluted area was observed for deaths from esophageal cancer (1.78), but the increase was not statistically significant. There was no significant difference in mortality from any type of cancer between men in the exposed and control areas. In women, however, the adjusted hazard ratio for colorectal cancer deaths was significantly higher in exposed subjects than in controls, while the hazard ratio for lung cancer deaths was lower with borderline significance.

To investigate the relationship between Cd exposure levels and cancer mortality, we divided the subjects living in the Cd-polluted area into the four groups with different exposure levels (none/borderline, mild, moderate, and high exposure) reported by Kawano (1996) [13] and analyzed the hazard ratios in the mild-, moderate-, and high-exposure groups compared with those in the none/borderline-exposure group after adjusting for effect-modifying factors (Table 5 and Table 6). Men in the high-exposure group had a lower adjusted hazard ratio for deaths from all malignant neoplasms (borderline significance) and for lung cancer deaths. The adjusted hazard ratios for stomach cancer deaths were significantly higher in the mild- and high-exposure groups, and higher (borderline significance) in the moderate-exposure group (Table 5). In women, the only significantly lower hazard ratio was observed for lung cancer deaths in the moderate-exposure group (Table 6).

### 3.3. Comparisons of Cancer Mortality between Exposed Residents with and without Cd-Induced Renal Damage

Renal effects, indicated by proteinuria, glucosuria, and glucoproteinuria, were associated with higher exposure to Cd (Table 3), but it is possible that the glucosuria group included patients with Type 2 diabetes who are at risk of developing malignancies. Therefore, we excluded 10 subjects (five men and five women) who showed increased fasting blood sugar more than 125 (mg/dL) in the second step of the baseline health screening test from the subjects for the mortality analysis. Then, we calculated the hazard ratios for subjects with urinary findings after adjustment, compared with ratios in subjects without urinary findings in the Cd-polluted area (Table 7).

In men, the lower adjusted hazard ratio for deaths from total malignant neoplasms was not significant, but the hazard ratio for lung cancer deaths was significantly lower in men with urinary findings. In contrast, the adjusted hazard ratio for cancer deaths of the kidneys and urinal tract was 2.39, but the increase in the hazard ratio was not significant in men. In women, adjusted hazard ratios for deaths from total malignant neoplasms and kidney and urinary tract cancers, particularly kidney cancer, were significantly higher in the Cd-exposed subjects with urinary findings. The hazard ratio for deaths from uterine cancer was also significantly higher in the subjects with urinary findings. The adjusted hazard ratio from pancreas cancer was higher (1.99), but its significance was borderline (*p* = 0.093).

## 4. Discussion

### 4.1. Cd Exposure and Cancer Mortality

In Cd-exposed women, the hazard ratio for deaths from colorectal cancer differed significantly from that of controls, although no dose–response relationship was found between Cd exposure level and hazard ratio for deaths from any malignant neoplasms. This lack of relationship suggests that women living in the Cd-polluted Jinzu River basin area are not at a greater risk of dying of cancer.

In men, the adjusted hazard ratios for stomach cancer deaths were significantly higher for subjects exposed to mild, moderate, and high levels of Cd. No dose–response relationship, however, was found between Cd exposure levels and risk of stomach cancer deaths. The hazard ratio for lung cancer deaths was significantly lower in men exposed to high levels of Cd who had a historical record of proteinuria, glucosuria, and glucoproteinuria, suggesting a possible inverse association between Cd exposure or Cd-induced renal damage and lung cancer.

In our previous 22-year follow-up study in residents of another Cd-polluted area, the Kakehashi River basin in Japan, no association was found between Cd exposure levels, as indicated by urinary Cd content, and deaths from stomach and lung cancer in men. In women, however, hazard ratios for lung cancer deaths were significantly lower in the subjects with urinary Cd ≥10 μg/g Cr than in subjects with Cd <10 μg/g Cr [15]. These findings suggest an inverse association between Cd exposure and lung cancer mortality, although the findings differed by sex.

Two meta-analyses of lung cancer risks associated with Cd exposure have been published, but their findings were inconsistent. Nawrot et al. (2015) analyzed three cohort studies and reported a significantly higher risk for lung cancer with environmental Cd exposure [16]. Chen et al. (2016) included environmental and occupational exposures, and could not find significant associations between Cd exposure and an increased risk of lung cancer [17].

### 4.2. Cd-Induced Renal Damage and Cancer Mortality

Renal tubular dysfunction is a characteristic symptom of chronic Cd poisoning and is indicated by glucoproteinuria (both proteinuria and glucosuria). We previously found increased cancer mortality in women with glucoproteinuria in the same cohort in the Jinzu river basin (Maruzeni et al. 2014). In the present analysis, we used proteinuria and/or glucosuria as a marker of Cd-induced renal effects. Their Cd exposure levels were remarkably high, with the 95th percentile urinary Cd ≥20 μg/L, suggesting Cd exposure from environmental pollution. Because the 95th percentile of urinary Cd was reported to be 3.50 μg/L in smokers aged ≥50 years old [18], this suggested that smoking alone, a major cause of Cd exposure in the general population, cannot increase Cd exposure levels. The prevalence of proteinuria, glucosuria, and glucoproteinuria increased as Cd exposure levels increased, suggesting that either proteinuria or glucosuria can also serve as indicators of Cd-induced renal damage. However, type 2 diabetes is a risk factor for developing cancers, and subjects with only glucosuria might include patients with type 2 diabetes. Therefore, we deleted subjects at a risk of diabetes who showed fasted blood sugar ≥125 (mg/dL) from the subjects with proteinuria, glucosuria, and glucoproteinuria and analyzed their mortality risk ratios compared with the subjects without urinary findings.

In the present analysis, the mortality risk from malignant neoplasms in women was significantly higher in Cd-exposed subjects who had a history of urinary findings of proteinuria, glucosuria, and glucoproteinuria. Deaths from kidney and urinary tract cancers, including renal cancer, uterine cancer, and pancreatic cancer, were observed in this cohort. These findings were not detected in our previous analysis, which used glucoproteinuria as a biomarker of renal effects [10]. The different results between our present and previous analysis suggest that in Cd-polluted areas, women who present with proteinuria, glucosuria, and glucoproteinuria may be at higher risk of death from kidney and urinary tract cancers, particularly renal cancer. In addition, we analyzed adjusted hazard ratios for deaths from renal and urinary tract cancer in a model including two factors—renal damage and exposure levels—in women. We found that only the renal damage factor was significantly associated with mortality risk. This finding suggests that Cd-induced renal damage may be a risk factor for death from renal cancer in women living in the Cd-polluted area.

Il’yasova and Schwartz (2005) performed a systematic review of studies on Cd exposure and renal cancer, and reported an increased risk in large-scale epidemiological studies with data from four countries [9]. Cd content in the kidney cortex is not an effective marker of Cd accumulation following renal cellular damage [19], and other markers should be used to evaluate exposure levels in cancer patients. Therefore, an association between Cd exposure and renal cancer could not be concluded from the findings in these clinical studies.

We reported an increased risk of mortality from uterine cancer in women with renal damage in our previous study [10], confirming the positive association between Cd exposure and death from uterine cancer observed in the present analysis. In the USA, increased mortality from uterine cancer was reported to be associated with increasing urinary Cd levels in a study using data from the Third National Health and Nutrition Examination Survey (NHANES III) [8]. These results suggest an increased mortality risk from uterine cancer is associated with increasing Cd exposure.

In the same mortality study by Adams et al. (2012), significantly increased mortality from pancreatic cancer associated with urinary Cd was reported in men [8]. In the meta-analysis of cohorts with highly Cd-exposed workers, Schwartz and Reis (2000) indicated there was an increased risk of pancreatic cancer with borderline significance (standardized mortality ratio = 166; *p* = 0.059) in both sexes [20]. In the present study, increased mortality from prostatic cancer was also of borderline significance, but only in women. A further follow-up study may be required to determine whether Cd exposure influences the development of pancreatic cancers.

In men with historical records of proteinuria, glucosuria, and glucoproteinuria, the adjusted hazard ratios for mortality from kidney and urinary tract cancers were higher, with borderline significance. However, no death from kidney cancer was found in men with urinary findings; thus, the effects of Cd-induced renal damage on renal cancer mortality may be limited in men. In addition, the adjusted hazard ratio for prostate cancer deaths was higher, but its increase was not significant. An increased prostate cancer risk has been reported in several follow-up surveys in industrial workers in Europe [3,4,21]. In our study in the Kakehashi River basin as well as in the present study, no association between renal damage and prostate cancer deaths was found [22]. This lack of association may be because the European studies measured the incidence of prostate cancer, while we only measured mortality and because their cohorts had occupational exposure, while the exposure in our study was environmental.

Mortality from lung cancer was significantly lower in Cd-exposed men with a historical record of urinary findings. Since a lower risk of mortality from lung cancer was found in the dose–response analysis, we performed additional calculations with an exposure factor and a renal-effect factor and found that both factors were independently associated, with borderline significance, with decreased mortality from lung cancer in men. In our previous study [10], we did not find differences in risk of mortality from lung cancer in Cd-exposed men with glucoproteinuria. Therefore, we cannot conclude that Cd-induced renal damage decreases the mortality risk from lung cancer.

### 4.3. Limitations

Our present study has several limitations that should be considered. Follow-up data from the subjects included mortality from cancers but not their incidence. Therefore, we could not estimate cancer risk, particularly for cancers with high survival rates, such as prostate, uterine, and breast cancers. Stomach cancer can be found and treated at early stages, suggesting that its incidence may be a more important indicator of risk than mortality. For renal cancer, however, mortality may be an adequate risk indicator, because renal cancer is difficult to diagnose at an early stage, and most cases are fatal.

Another limitation is that environmental Cd exposure levels were based on the contribution of contaminated Jinzu River water [13], and not biological markers of Cd body burden, such as urinary levels in each subject, which are more closely related to renal effects. In addition, we used historical exposure levels from the baseline survey, and did not measure Cd exposure during the follow-up period. Cd body burden, however, may not have significantly increased during the follow-up period, because ingestion of Cd-polluted rice and river water was prohibited in the Jinzu River basin, and residents are presumed to have had no additional Cd exposure during that period.

We used proteinuria, glucosuria, and glucoproteinuria as markers of renal damage in this analysis, but these urinary findings are not specific to renal tubular dysfunction. We first analyzed the dose–response relationship between Cd exposure levels and the prevalence of proteinuria and/or glucosuria to confirm that this marker indicated renal effects associated with Cd exposure. Urinary Cd levels and urinary β2-MG positive rates of the subjects with proteinuria, glucosuria, and glucoproteinuria were high enough to develop renal effects. Moreover, the subjects who showed glucosuria (without proteinuria) and diabetic elevation of blood sugar in the second step of the baseline health screening test were excluded from the mortality analysis to avoid confounding by the presence of type 2 diabetes. After these modifications, we believe that proteinuria, glucosuria, and glucoproteinuria can be used as markers of renal damage in these subjects living in a heavily contaminated area to Cd in Japan.

## 5. Conclusions

Our findings suggest that women with renal damage associated with high Cd exposure levels are at risk of mortality from malignant neoplasms, including renal and uterine cancers. Mortality risk from pancreatic cancer may be increased in women with Cd-induced renal damage, but more studies are required to confirm this hypothesis.

## Figures and Tables

**Table 1:** Age distribution of prevalence of urinary findings: positive cases, smokers and cases with a hypertensive history.

Sex	Age	No (%)	Pro(+)/Glu(+)(%)	Smoking (%)	HT (%)	No (%)	Pro(+)/Glu(+)(%)	Smoking (%)	HT (%)
		Control				Exposed			
Men	50–59	383 (41.4)	67 (17.5)	267 (69.7)	80 (20.9)	1443 (42.9)	283 (19.6)	1028 (71.4)	242 (16.8)
	60–69	318 (34.3)	66 (20.8)	186 (58.7)	79 (24.8)	1106 (32.9)	247 (22.3)	674 (61.2)	263 (23.8)
	70–79	179 (19.3)	36 (20.1)	85 (47.5)	60 (33.5)	623 (18.5)	197 (31.6)	319 (51.4)	178 (28.6)
	≥80	46 (5.0)	9 (19.6)	16 (34.8)	10 (21.7)	191 (5.7)	80 (41.9)	56 (29.8)	53 (27.7)
	Total	926 (100.0)	178 (19.2)	554 (59.9)	229 (24.7)	3363 (100.0)	807 (24.0)	2077 (62.0)	736 (21.9)
Women	50–59	474 (40.4)	37 (7.8)	9 (1.9)	97 (20.5)	1613 (40.5)	152 (9.4)	94 (5.8)	254 (15.7)
	60–69	372 (31.7)	41 (11.0)	8 (2.2)	102 (27.4)	1289 (32.3)	237 (18.4)	119 (9.3)	308 (23.9)
	70–79	231 (19.7)	37 (16.0)	5 (2.2)	69 (29.9)	883 (22.2)	304 (34.4)	80 (9.1)	231 (26.2)
	≥80	95 (8.1)	18 (18.9)	6 (6.4)	28 (29.5)	200 (5.0)	108 (54.0)	19 (9.6)	54 (27.0)
	Total	1172 (100.0)	133 (11.3)	28 (2.4)	296 (25.3)	3985 (100.0)	801 (20.1)	312 (7.9)	847 (21.3)

Note, missing 1 control man, 13 exposed men, 4 control women and 11 exposed women from the smoking group, Pro(+)/Glu(+): proteinuria, glucosuria, and glucoproteinuria, No: number of subjects, HT: hypertension history.

**Table 2:** Median and 90th and 95th percentiles of urinary Cd and positive rate (%) of urinary β2-microglobuline (≥1 mg/dL) in groups with different urinary findings.

Sex	Urinary Findings	N	Urinary Cd (μg/L)			Urinary β2-MG(+)	
			Median	90th per.	95th per.	N	%
Men	Glu(+) and Pro(−)	495	9.1	25.0	38.0	62	12.5
	Pro(+) and Glu(−)	149	10.0	27.0	45.0	40	26.8
	Pro(+) and Glu(+)	159	12.0	22.0	28.9	120	75.5
	Pro(+)/Glu(+)	803	10.0	24.0	34.9	222	27.6
Women	Glu(+) and Pro(−)	395	8.5	20.0	28.0	145	36.7
	Pro(+) and Glu(−)	136	8.5	19.2	37.7	34	25.0
	Pro(+) and Glu(+)	267	7.7	17.9	22.0	242	90.6
	Pro(+)/Glu(+)	793	8.3	19	22.9	421	53.1

Note, N: number of subjects, per.: percentile, β2-MG(+): beta2-microglobuline positive (≥1 mg/dL), Glu(+): glucosuria, Pro(+): proteinuria, Pro(+)/Glu(+): proteinuria, glucosuria, and glucoproteinuria.

**Table 3:** Prevalence of renal damage indicated by proteinuria, glucosuria, and glucoproteinuria and Cd exposure.

Analysis	Sex	Exposure	No	Pro(+)/Glu(+) (%)	Odds Ratio	(95%CI)	*p*
Model 1	Men	Control	926	178 (19.2)	1.00		
		Exposed	3363	807 (24.0)	1.32	(1.10, 1.59)	0.003
Model 2	Men	Controls	926	178 (19.2)	1.00		
		Non/Boderline	1040	186 (17.9)	0.92	(0.74, 1.16)	0.501
		Mild	1231	275 (22.3)	1.21	(0.98, 1.49)	0.085
		Moderate	515	115 (22.3)	1.18	(0.90, 1.53)	0.236
		High	577	231 (40.0)	2.83	(2.24, 3.59)	0.000
Model 1	Women	Control	1172	133 (11.3)	1.00		
		Exposed	3985	801 (20.1)	2.21	(1.80, 2.72)	0.000
Model 2	Women	Controls	1172	133 (11.3)	1.00		
		Non/Boderline	1228	148 (12.1)	1.13	(0.87, 1.47)	0.347
		Mild	1508	258 (17.1)	1.82	(1.44, 2.30)	0.000
		Moderate	580	129 (22.2)	2.67	(2.02, 3.54)	0.000
		High	669	266 (39.8)	6.20	(4.81, 7.99)	0.000

Note, covariates of logistic analysis: age, HT (hypertension) history, smoking, No: number of subjects, Pro(+)/Glu(+): proteinuria, glucosuria, and glucoproteinuria, CI: confident interval, *p*: *p*-value.

**Table 4:** Mortality risk ratios for malignant neoplasms of Cd-exposed subjects compared with controls.

Sex	Cancer	D	HR	D	HR	95%CI
		Controls		Exposed		
		**(No = 926)**		**(No = 3363)**		
Men	Total	174	1.00	653	0.98	0.8, 1.2
	Esophagus	4	1.00	27	1.78	0.6, 5.1
	Stomach	57	1.00	180	0.83	0.6, 1.1
	Colon, rectum	16	1.00	64	1.05	0.6, 1.8
	Liver	16	1.00	58	0.96	0.6, 1.7
	Gallbladder	7	1.00	25	0.94	0.4, 2.2
	Pancreas	9	1.00	41	1.15	0.6, 2.4
	Lung	37	1.00	143	1.01	0.7, 1.5
	Prostate	5	1.00	21	1.05	0.4, 2.8
	Bladder	3	1.00	13	1.09	0.3, 3.8
	Kidney/urinal tract	2	1.00	8	1.09	0.2, 5.1
	(Kidney)	0	-	0	-	-
	Lymph/blood forming organs	7	1.00	31	1.15	0.5, 2.6
		**(No = 1172)**		**(No = 3985)**		
Women	Total	114	1.00	437	1.05	0.9, 1.3
	Esophagus	0	-	4	-	-
	Stomach	33	1.00	98	0.83	0.6, 1.2
	Colon, rectum	8	1.00	66	2.28	1.1, 4.8 *
	Liver	5	1.00	27	1.43	0.5, 3.7
	Gallbladder	8	1.00	46	1.62	0.8, 3.4
	Pancreas	14	1.00	34	0.67	0.4, 1.3
	Lung	19	1.00	44	0.63	0.4, 1.1
	Breast	4	1.00	14	0.92	0.3, 2.8
	Uterus	3	1.00	15	1.44	0.4, 5.0
	Ovary	2	1.00	9	1.31	0.3, 6.1
	Bladder	2	1.00	4	0.62	0.1, 3.4
	Kidney and urinal tract	1	1.00	8	2.16	0.3, 17.4
	(Kidney)	0	-	4	-	-
	Lymph/blood-forming organs	5	1.00	27	1.39	0.5, 3.6

Note, No: number of subjects, D: number of deaths, HR: hazard ratio, CI: confident interval, *: *p* < 0.05.

**Table 5:** Mortality risk ratios for cancer in three exposed male groups compared with men living in the areas with borderline exposure in the Cd-exposed Jinzu River basin.

Mortality	D	HR	D	HR	95%CI	D	HR	95%CI	D	HR	95%CI
Exposure Levels	Non/Border		Mild			Moderate			High		
No	1040		1231			515			577		
Total	202	1.00	251	1.02	0.84, 1.23	107	1.12	0.88, 1.42	93	0.79	0.61, 1.01 #
Esophagus	13	1.00	9	0.62	0.26, 1.48	4	0.53	0.15, 1.90	1	0.16	0.02, 1.23
Stomach	39	1.00	76	1.58	1.06, 2.36	29	1.62	0.99, 2.64	36	1.61	1.00, 2.57 *
Colon, rectum	22	1.00	24	0.90	0.50, 1.62	10	1.02	0.48, 2.17	8	0.61	0.26, 1.45
Liver	20	1.00	20	0.82	0.44, 1.52	5	0.42	0.14, 1.23	13	1.08	0.52, 2.21
Gallbladder	8	1.00	6	0.58	0.20, 1.68	7	1.77	0.64, 4.90	4	0.79	0.24, 2.63
Pancreas	12	1.00	17	1.11	0.53, 2.32	7	1.19	0.47, 3.02	5	0.54	0.17, 1.67
Lung	50	1.00	58	0.96	0.65, 1.42	21	0.91	0.54, 1.52	14	0.53	0.29, 0.96 *
Prostate	7	1.00	6	0.79	0.25, 2.46	4	1.30	0.37, 4.63	4	0.79	0.20, 3.19
Bladder	5	1.00	4	0.63	0.17, 2.36	3	1.18	0.28, 4.95	1	0.34	0.04, 2.90
Kidney/urinal tract	2	1.00	3	2.22	0.23, 21.3	2	3.79	0.34, 41.9	1	1.56	0.10, 25.2
(Kidney)	0	-	0	-	-	0	-	-	0	-	-
Lymph/blood-forming organs	10	1.00	12	0.97	0.42, 2.25	5	1.00	0.34, 2.94	4	0.67	0.21, 2.16

Note, No: number of subjects, D: number of deaths, HR: hazard ratio, CI: confident interval, #: *p* < 0.1, *: *p* < 0.05.

**Table 6:** Mortality risk ratios for cancer in three exposed female groups compared with women living in the areas with borderline exposure in the Cd-exposed Jinzu River basin.

Mortality	D	HR	D	HR	95%CI	D	HR	95%CI	D	HR	95%CI
Exposure levels	Non/Border		Mild			Moderate			High		
No	1228		1508			580			669		
Total	144	1.00	167	0.98	0.77, 1.23	56	0.84	0.61, 1.16	70	0.99	0.74, 1.34
Esophagus	1	1.00	1	-	-	1	-	-	1	-	-
Stomach	32	1.00	33	0.91	0.55, 1.51	9	0.66	0.31, 1.40	24	1.50	0.87, 2.62
Colon, rectum	24	1.00	21	0.72	0.40, 1.30	10	0.91	0.43, 1.92	11	0.89	0.43, 1.84
Liver	7	1.00	13	1.90	0.67, 5.41	4	1.16	0.28, 4.85	3	0.73	0.14, 3.75
Gallbladder	11	1.00	21	1.43	0.64, 3.20	9	2.01	0.80, 5.07	5	1.07	0.36, 3.21
Pancreas	12	1.00	12	0.88	0.39, 2.01	4	0.59	0.17, 2.13	6	1.10	0.40, 2.99
Lung	19	1.00	19	0.90	0.47, 1.72	1	0.12	0.02, 0.90 *	5	0.48	0.16, 1.43
Breast	5	1.00	3	0.56	0.12, 2.49	5	2.32	0.62, 8.65	1	0.45	0.05, 4.02
Uterus	5	1.00	6	1.25	0.30, 5.25	1	0.66	0.07, 6.35	3	1.83	0.37, 9.14
Ovary	5	1.00	2	0.30	0.06, 1.53	1	0.40	0.05, 3.39	1	0.39	0.05, 3.36
Bladder	1	1.00	3	2.35	0.24, 22.8	0	-	-	0	-	-
Kidney/urinal tract	1	1.00	4	3.03	0.34, 27.2	2	4.10	0.37, 45.3	1	1.97	0.12, 31.7
(Kidney)	1	1.00	2	1.58	0.14, 1.75	0	-	-	1	2.30	0.14, 38.2
Lymph/blood-forming organs	9	1.00	8	0.70	0.27, 1.83	5	0.91	0.28, 2.97	5	1.07	0.36, 3.20

Note, No: number of subjects, D: number of deaths, HR: hazard ratio, CI: confident interval, *: *p* < 0.05.

**Table 7:** Adjusted hazard ratios for cancer in subjects with proteinuria, glucosuria, and glucoproteinuria, compared with subjects without urinary findings living in the exposed area.

Sex	Cancer	D	HR	D	HR	95%CI
		No Findings		Proteinuria and/or Glucosuria		
		**(No = 2556)**		**(No = 802)**		
Men	Total	522	1.00	130	0.87	0.71, 1.06
	Esophagus	20	1.00	7	1.13	0.45, 2.86
	Stomach	142	1.00	38	0.95	0.66, 1.37
	Colon, rectum	53	1.00	10	0.73	0.37, 1.43
	Liver	42	1.00	16	1.44	0.80, 2.59
	Gall bladder	21	1.00	4	0.68	0.23, 1.98
	Pancreas	33	1.00	8	0.91	0.42, 1.98
	Lung	121	1.00	22	0.62	0.39, 0.98 *
	Prostate	13	1.00	8	1.59	0.60, 4.25
	Bladder	11	1.00	2	0.59	0.13, 2.71
	Kidney/urinal tract	5	1.00	3	2.39	0.52, 10.9
	(Kidney)	0	1.00	0	-	-
	Lymph/blood-forming organs	24	1.00	7	1.02	0.44, 2.39
		**(No = 3184)**		**(No = 796)**		
Women	Total	339	1.00	97	1.49	1.17, 1.89 *
	Esophagus	2	1.00	2	1.82	0.15, 21.7
	Stomach	77	1.00	21	1.19	0.71, 1.98
	Colon, rectum	55	1.00	11	0.87	0.44, 1.70
	Liver	24	1.00	3	0.60	0.14, 2.61
	Gallbladder	36	1.00	10	1.75	0.84, 3.65
	Pancreas	25	1.00	9	1.99	0.89, 4.44 #
	Lung	35	1.00	9	1.40	0.65, 3.00
	Breast	11	1.00	3	2.21	0.59, 8.28
	Uterus	10	1.00	5	3.85	1.16, 12.8 *
	Ovary	7	1.00	2	1.58	0.31, 8.02
	Bladder	4	1.00	0	-	-
	Kidney and urinal tract	3	1.00	5	10.1	2.29, 44.5 *
	(Kidney)	2	1.00	2	7.71	1.05, 56.8 *
	Lymph/blood-forming organs	20	1.00	6	1.98	0.77, 5.09

Note, No (urinary) findings: nether proteinuria nor glucosuria, No: number of subjects, D: number of deaths, HR: hazard ratio, CI: confident interval, #: *p* < 0.1, *: *p* < 0.05.
